# Misfolding of Amyloidogenic Proteins and Their Interactions with Membranes

**DOI:** 10.3390/biom4010020

**Published:** 2013-12-27

**Authors:** Annalisa Relini, Nadia Marano, Alessandra Gliozzi

**Affiliations:** 1 Department of Physics, University of Genoa, Genoa 16146, Italy; E-Mails: relini@fisica.unige.it (A.R.); nmarano@stlawu.edu (N.M.); 2 Research Centre on the Molecular Basis of Neurodegeneration, Florence 50134 Italy; 3 Department of Chemistry, Saint Lawrence University, Canton, NY 13617, USA

**Keywords:** amyloidogenic proteins, misfolding, amyloid aggregation, fibrillogenesis, membrane permeabilization, amyloid toxicity

## Abstract

In this paper, we discuss amyloidogenic proteins, their misfolding, resulting structures, and interactions with membranes, which lead to membrane damage and subsequent cell death. Many of these proteins are implicated in serious illnesses such as Alzheimer’s disease and Parkinson’s disease. Misfolding of amyloidogenic proteins leads to the formation of polymorphic oligomers and fibrils. Oligomeric aggregates are widely thought to be the toxic species, however, fibrils also play a role in membrane damage. We focus on the structure of these aggregates and their interactions with model membranes. Study of interactions of amlyoidogenic proteins with model and natural membranes has shown the importance of the lipid bilayer in protein misfolding and aggregation and has led to the development of several models for membrane permeabilization by the resulting amyloid aggregates. We discuss several of these models: formation of structured pores by misfolded amyloidogenic proteins, extraction of lipids, interactions with receptors in biological membranes, and membrane destabilization by amyloid aggregates perhaps analogous to that caused by antimicrobial peptides.

## 1. Introduction

Several serious diseases, including Alzheimer’s, Parkinson’s, and Type II diabetes mellitus are characterized by defects in the folding of specific proteins, leading to protein aggregation and the appearance of amyloid deposits. These diseases are marked by the deposition of amyloid fibrils and plaques in various tissues of affected patients. Thus the misfolding and aggregation of these proteins is a key step in the development of these disorders.

It has been proposed that the ability to form amyloid aggregates is a generic property of the polypeptide chain, as the amyloid aggregate structure is based on β-sheets stabilized by hydrogen bonds which involve the polypeptide backbone [1]. The hypothesis of amyloid as a generic protein fold has been reinforced by the discovery of a number of amyloid structures associated with normal physiologic conditions, not only in simple organisms such as bacteria, fungi or insects but also in humans [2,3,4]. We will focus on pathological amyloid, although structures and assembly of all amyloids have similarities. Amyloid aggregation is a nucleation-dependent process, usually characterized by the presence of a lag phase followed by an exponential growth phase. Protein monomers first self-assemble into oligomers, followed by assembly into more complex structures. Despite similarities, the process leading to oligomer and fibril formation is strongly polymorphic. A variety of intermediate structures have been described depending on the protein and on the aggregation conditions. In addition, polypeptide chains can access different spatial configurations within the aggregates, resulting in structural degeneracy.

In order to aggregate, amyloid-forming globular proteins generally go through a partially unfolded intermediate [5,6]. Complete unfolding does not appear to be necessary, but some structural flexibility is needed in order for aggregation to occur (see [7] and references therein). In the case of intrinsically disordered proteins, more structured forms appear to be the species involved in aggregation [8,9].

Surfaces, especially lipid membranes, can catalyze misfolding and amyloid aggregation [10,11,12]. In addition, increasing evidence indicates that the interaction of amyloid aggregates with membranes is critical in the onset of amyloid diseases [13,14,15]. It is commonly accepted that the interaction between amyloid aggregates and membranes results in disruption of the barrier function of the membrane followed by intracellular calcium disregulation and oxidative stress [10]. These interactions can even change synaptic plasticity and induce neuronal cell death [16,17,18]. In most cases, oligomers, rather than mature fibrils, are the toxic species that disrupt the membrane permeability. However, in some cases, mature fibrils may also induce cell damage disassembling the membrane lipids [19].

Although many models have been proposed, the molecular mechanisms for toxicity have not been entirely solved. In this review we discuss misfolding of proteins leading to amyloid aggregation, structure of amyloid aggregates, and present recent models and experimental evidence of several mechanisms that might be active in amyloid toxicity.

## 2. Protein Unfolding and Misfolding

### 2.1. Amyloid Misfolding, Aggregation and Protein Conformation

Aggregation is a complex process that can occur in different ways, often through a partially unfolded intermediate [5,6] but it can also occur from native-like conformations [7]. Studies on the model protein HypF-N (the N-terminal domain of the hydrogenase maturation factor HypF from *Escherichia coli*) have shown that under conditions that promote aggregation, the protein is in a partially unfolded pre-molten globule state [20]. However, aggregation is not promoted by the least ordered regions but by particular aggregation-prone sequences, such as those having a high hydrophobicity [21]. In addition, oligomers may form where the protein retains a native-like secondary structure, later converting to more typical amyloid structures with a high β-sheet content, as has been shown for insulin [22], S6 from *Thermus thermophilus* [23], and acylphosphatse from *Sulfolobus solfataricus* [24]. Under aggregating conditions, proteins have a more flexible native structure than under non-aggregating conditions [23,25]. Thus, it appears that at least some structural flexibility is needed in order for aggregation to occur. The typical cross-β amyloid structure may form after the initial protein aggregation.

Several proteins implicated in amyloid disease, for example Aβ in Alzheimer’s disease, amyloid islet protein in Type II diabetes (hIAPP), and α-synuclein in Parkinson’s disease, are intrinsically disordered proteins (IDPs) [26,27,28]. IDPs do not have a dominant stable native structure as do globular proteins, but have a high content of random coil with variable amounts of secondary and tertiary structure, and may sample many transient conformational states, generally acquiring a more defined structure upon binding to ligands/binding partners (for review see [8]). The transient conformations of IDPs have been studied experimentally [29,30] and using simulations [29,31]; these studies have found that some areas of the protein chain are more structured than others. Like globular proteins, IDPs also appear to aggregate from a semistructured (rather than fully unstructured) state [6,32,33]. Qiao *et al*. have proposed that for hIAPP, some of the IDP metastable states can act as fibrillization nuclei [31]. Jain *et al*. have suggested that at high protein concentrations, collapsed forms (premolten globule-like) of IDPs that form under physiological conditions can then interact and form aggregates [32]. In regions that particularly contribute to the amyloidogenicty of IDPs, structure-promoting amino acid residues tend to increase the propensity for forming amyloid structures [34]. Using structure predictions programs, Zhang *et al*. have suggested that semi-disordered regions of IDPs tend to be those involved in aggregation [9]. Studies on Aβ provide an illustrative example. Comparing the more aggregation prone Aβ(1–42) to Aβ(1–40), the former is more rigid [35], contains more β structure and more exposed hydrophobic patches under non-denaturing conditions [36]. These features disappear under denaturing conditions where fibers form less readily [36]. More recent work has also implicated increased structure, specifically a β-hairpin in the C-terminal region in aggregation of Aβ(1–42) [29,37]. All this evidence points to the likelihood of partially structured regions being important in amyloid aggregation of IDPs. Aggregation may stabilize partially folded states in IDPs, for example α-helices for α-synuclein, which then convert to sheets [6].

**Figure 1:**
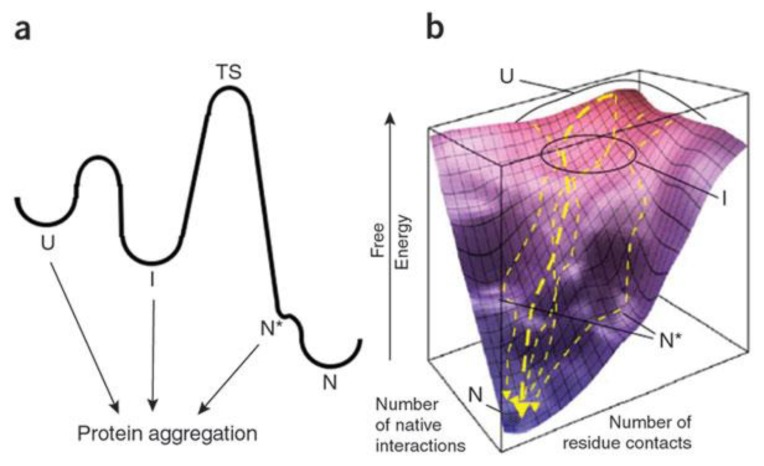
Aggregation can occur from different protein conformations (**a**) kinetic profile; (**b**) thermodynamic energy landscape. U = unfolded, I = intermediate, N = native, N* = native-like locally unfolded state with low free energy barrier, TS = transition state. Reprinted by permission from Macmillan Publishers Ltd.: *NATURE CHEMICAL BIOLOGY*, Chiti, F.; Dobson, C.M. Amyloid formation by globular proteins under native conditions, ***5***, 15–22. copyright 2009 [7].

Since even globular protein conformations are not static and many possible conformations may exist, one can look at energy landscapes to provide insight into misfolding. Although fully unfolded proteins can aggregate, some conformations, which are more native-like and not separated by a large free energy barrier (illustrated as N* in Figure 1) may be of particular importance to aggregation under more physiological conditions [7].

Detailed experiments comparing acylphosphatase under conditions that do not result in aggregation and with 5%TFE, which induces aggregation, showed that although the thermodynamic stability of the protein was similar, under aggregation conditions some backbone amides were more exposed [38]. Comparison of energy landscapes revealed some conformations that were less native-like and exposed more hydrophobic surface. The free energy barrier between the native and aggregation-prone states is lower in aggregating conditions thus the protein is more likely to access them [38]. Interestingly, in the presence of bound ligand, which decreases the propensity for aggregation, there are reductions in backbone dynamics, and energy landscapes show that aggregation-prone conformations are less accessible [38].

Unlike globular proteins, the energy landscapes of IDPs do not show one deep local minimum but are much shallower with many minima without intrinsic large energy barriers [8]. Thus these proteins can easily sample many conformations. Environmental conditions and binding partners can stabilize some conformations [8]. Recent work combining molecular dymamics simulations and nuclear magnetic resonance (NMR) found a wide variety of ensembles in the landscape of Aβ. However, conformations with a C-terminal β-hairpin were more common in Aβ(1–42) in contrast to the less aggregation-prone Aβ(1–40) and could serve as sites for aggregation [29].

**Figure 2:**
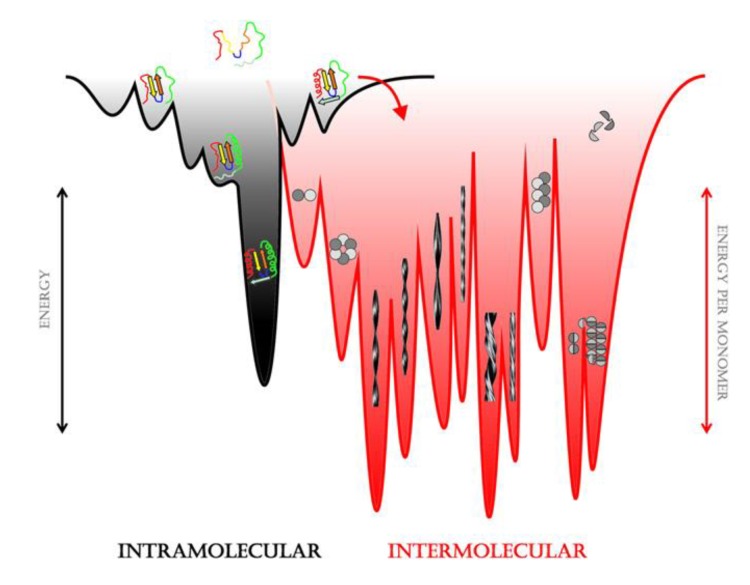
Representative sketch of energy landscapes. Folding intermediates can connect the folding (at left in black) and aggregation energy landscapes (at right in red). Extensive polymorphism can exist in both oligomeric and fibrillar states. Reprinted from *Molecular Cell*, **43**, Eichner, T.; Radford, S.E., 8–18, A diversity of assembly mechanisms of a generic amyloid fold. Copyright 2011 with permission from Elsevier [44].

Specific intermediates in the folding pathways of globular proteins can also be responsible for aggregation. Studies on wild-type and mutant lysozyme found in lysozyme amyloidosis showed that amyloid fiber formation was most prominent under conditions where there was a high population of partially folded states (as compared to folded or fully unfolded) [39]. Both wild-type and mutant lysozyme underwent similar structural changes; however, only in the mutant were the partially folded states populated to any significant extent under physiological conditions [39]. Interestingly, recent studies have shown that glycation of lysozyme by prolonged incubation with concentrations of glucose that can be present in diabetic conditions also leads to partial unfolding and amyloid aggregation [40]. Kinetic studies on HypF-N have shown that under slightly destabilizing conditions, where the protein is still predominantly in its native state, the population of molecules in a partially folded state increases, and it is this state that is responsible for increased aggregation under these conditions [41]. Structural studies on the folding mechanism of β2-microglobulin, which is responsible for dialysis-related amyloidosis, have found a long-lived folding intermediate that contains a non-native *trans*-proline residue to be the likely amyloid precursor [42]. A recent study using a variety of biophysical methods has provided experimental evidence that this intermediate is much more likely to aggregate than the native state [43]. The authors point out that a few percent of the molecules in a native sample can be in non-native conformations that are higher in free energy by a few kcal/mol. These partially folded intermediates can thus act as links between folding and aggregation landscapes [7,44] as illustrated in Figure 2.

The picture that emerges is that globular proteins may aggregate from unfolded, partially folded or near native states as seen in Figure 1 [7]. Conditions leading to misfolding can be seen as changing the landscape allowing easier access to aggregation-prone states. On the other hand, IDPs appear to aggregate from their more structured states [8].

### 2.2. Factors Contributing to Unfolding/Misfolding

The formation of amyloid fibers can be promoted by a variety of environmental conditions, for example increased temperature [45,46,47], low pH [45,46,48], organic solvents [46], agitation/shear [47,48,49,50,51,52]. These conditions tend to destabilize the native structure of globular proteins and can expose aggregation-promoting hydrophobic residues. Many types of ions can also affect the propensity of proteins to undergo amyloid misfolding and aggregation [53,54,55,56]. Water-ordering ions (kosmotropes), which would increase the hydrophobic effect, tend to increase the rate of aggregation, whereas chaotropic ions have the opposite effect [56,57]. As we have seen in the last section, in the case of IDPs, aggregation occurs from semi-structured states. IDPs may gain structure under conditions where globular proteins lose it [8]. Thus conditions that favor conformational states that are involved in self-association would tend to promote aggregation. For example, α-synuclein was found to convert from its unfolded form to a partially folded intermediate that is the precursor to fibril formation in the presence of low concentrations of a variety of alcohols and fluorinated alcohols [58]. Under more physiological conditions, only a small amount of destabilization of globular proteins is likely to be necessary as even a small percentage of molecules present in a partially folded state can greatly enhance amyloid misfolding and aggregation rates [41,43]. Mutations and surfaces can increase the population of aggregation-prone states.

Although amyloid formation is a general property of polypeptides, amino acid sequence plays a role in the propensity of proteins to aggregate [1]. Thus, mutations can contribute to the propensity of proteins to misfold and form amyloid structures. For example, some of the mutations involved in early onset Alzheimer’s disease are in the Aβ peptide, increasing its aggregation propensity [59]. Many studies have looked at how mutations increase aggregation and several mechanisms appear to be responsible. Destabilization of the native structure is one possible mechanism. Early kinetic studies on lysozyme mutants implicated in a systemic inherited amyloidosis have shown that the stability of the native state relative to folding intermediates is not as great in the mutant relative to the wild type [60]. A more recently discovered mutation in β2-microglobulin leading to a systemic amyloidosis was found to reduce the stability of the native structure relative to wild type β2-microglobulin [52,61]. A truncation variant (∆N6) of β2-microglobulin, which is found in *in vivo* fibrils, is much more amyloidogenic and less stable than the full-length protein [62,63]. This variant was found to have a structure similar to the previously described folding intermediate thought to be the amyloidogenic species [64]. In addition, small amounts of this variant can nucleate aggregation of the full-length protein [65]. Mutations that increase hydrophobicity [66,67] or decrease charge [67] increase the rate of misfolding to amyloid structures; both these situations make it more likely that protein molecules will interact with each other and aggregate. Mutations that increase the likelihood of the sequence to adopt a β-sheet structure, not surprisingly, also increase amyloid misfolding [68]. Using random mutagenesis on residues 41 and 42 of Aβ(1–42), Kim and Hecht found a strong correlation between aggregation and both hydrophobicity and propensity to adopt a β-sheet structure [69].

An important class of mutations involving amyloid aggregation is that resulting in glutamine repeats leading to several degenerative diseases whose severity increases as the number of glutamines increases [70,71,72,73]. The increase in the number of uninterrupted glutamine residues (variable but beyond about 37) is correlated with an increased propensity of the protein to aggregate, eventually leading to disease [71,74]. Interestingly, modeling by Zhang *et al*. has shown that when the number of glutamines in the tracts is small (<24) these regions are fully disordered, but more and more residues become semi-disordered as the number of glutamines increases [9]. The length at which about one third of the glutamines is in the semi-disordered state correlates with the onset of pathogenicity providing support for the idea that aggregation occurs from a semi-disordered state.

Surfaces of various types have been implicated in accelerating amyloid fibrillization. Interactions of proteins with surfaces, which may be hydrophobic or contain charges, can lead to changes in folding of proteins favoring aggregation. In addition, surfaces are two-dimensional, thus they can increase effective local concentrations raising the probability of protein-protein interaction. Using surfaces of varying hydrophobicity as well as lipid bilayers, Shen *et al*. have shown that, in the presence of surfaces, Aβ is capable of amyloid misfolding and fibrillization at concentrations much lower than those required in bulk, as long as the surface allows monomers to bind and remain mobile to allow for two-dimensional diffusion and interaction [75]. There is a large body of literature describing experiments and simulations with amyloid-forming proteins and surfaces. We will give a few illustrative examples.

Because of the propensity of hydrophobic amino acid side chains to interact with each other in water, changes in protein folding that expose hydrophobic residues can accelerate protein aggregation. The air-water interface is one example, where proteins can unfold with hydrophobic residues exposed to the air, which can lead to misfolding and aggregation [54,76,77]. Hydrophobic surfaces can have similar effects. Nault *et al*. used a variety of biophysical techniques to study conformational changes in insulin when interacting with hydrophobic self-assembled monolayers [78]. They found that the hydrophobic surfaces accelerated the aggregation of insulin resulting in a final conformation similar to that seen in pH and temperature-induced aggregation. However they found adsorbed intermediates that were different in conformation from both native and fibrillar insulin, implying that a complex process is involved. Recent work on the globular JD domain of ataxin-3 showed that a hydrophobic gold surface but not a polar mica surface resulted in filament formation, although both surfaces caused some unfolding of the native structure (less with the mica substrate) [79]. In addition, the authors did molecular dynamics modeling of this system showing a much larger area of the protein in contact with the gold surface and unmasking of hydrophobic patches as compared with the protein in contact with mica. However, amyloid misfolding and aggregation can occur on polar surfaces as well depending on the protein (for example, the amyloidogenic light chain variable domain [80,81]).

For misfolding in organisms, biological surfaces are of particular interest as these can directly contribute to the misfolding that leads to pathologies [82]. Extracelluar matrix components can play such a role. Collagen binds β2-microglobulin and this binding may serve to trigger fibrillization and accumulation in the joints in dialysis related amyloidosis [83,84]. Studying the aggregation of β2-microglobulin, Relini *et al*. have shown that interaction with collagen fibers accelerates fibril formation under physiological conditions. They hypothesized that this was due to interaction of β2-microglobulin with positive charges on collagen, supported by experiments using positively charged poly-L-lysine, which also catalyzed fibril formation [85]. Interestingly, Bertoletti *et al*. were able to separate a folding intermediate thought to be the amyloidogenic species from the native form by ion exchange chromatography due to the greater exposure of negative charge on the intermediate [86]. Myers *et al*. also found an enhancing effect of collegen on β2-microglobulin fibrillogenesis [87]. Nascent collagen binds and induces fiber formation in amyloidogenic immunoglobulin light chains [88]. Other extracelluar matrix components such as proteoglycans and gycosaminoglycans have also been shown to accelerate β2-microglobulin amyloid fiber formation [87,89,90,91]. Proteoglycans and other basement membrane components are found in all types of amyloid deposits and promote fibrillogenesis (reviewed in [92,93,94].) For example, proteoglycans have been been found to bind and enhance fibril formation and stability of hIAPP [95,96,97] and Aβ [98,99]. Some early studies suggested that some basement membrane components could inhibit Aβ fibril formation [100,101,102,103]. However, a recent study found that basement membrane components (including collagen, fibronection, laminin) conjugated to sepharose beads accelerated fibril formation of Aβ on these surfaces in a model of cerebral amyloid angiopathy [104]. These authors suggest that the conflicting results may be due to the different conditions used in the later study (e.g., lower concentration, absence of an air-water interface).

Especially important to amyloid formation under physiological conditions are lipid membranes, as these are ubiquitous in living organisms. Lipid membranes have been shown to catalyze misfolding and aggregation and are also the sites for damage inflicted by aggregated proteins (reviewed in [11,12,13,105]). Interactions with membranes can affect protein conformation. In the case of α-synuclein, interactions with lipids cause both an increase in α-helical content and aggregation [106,107]. The presence of membranes appears to make the helical conformation more stable. Single molecule studies have shown that in the presence of lipids, the energy landscape of α-synuclein acquires two local minima corresponding to two α-helical conformations, the favored one depending on the concentration of the lipid and curvature of the vesicles [108]. Similar results were found for hIAPP; binding to membranes containing anionic lipids led to conversion to α-helical form and to eventual formation of β-sheets and fibrillization [109,110,111]. More recent detailed studies on the interactions of hIAPP with membranes have shown that the initially monomeric helical form is found within the lipid headgroup region, but once a sufficiently high protein concentration is reached, a transition to β-aggregates occurs. These are, however, found only on the surface of membrane [112].

Anionic lipids appear to be especially important in membrane-assisted conformational change, and electrostatic interactions may play a role in the protein-lipid interactions. The earlier described studies on α-synuclein and hIAPP showed that anionic lipids such as phosphatidylserine were required for protein binding [106,107,109,110,111]. Studies by Zhao *et al*. showed that a variety of different proteins formed fibrils in the presence of liposomes containing phosphatidylserine [10]. Molecular dynamics studies point to the importance of electrostatic interactions between anionic lipids and positively charged residues on the initial interactions of hIAPP with membranes [113]. These simulations showed that hydrophobic residues in the helix were exposed to solvent providing a hydrophobic surface that could initiate aggregation. The authors propose that this aggregation then leads to conversion to the β-sheet conformation [113] consistent with the studies of Lee *et al*. [114]. Similarly, studies on Aβ have generally shown that anionic lipid surfaces favor binding and eventual conversion to a β-conformation [115,116]. However, the situation is complex, as the exact membrane composition exerts an effect [117].

The lipid composition of natural membranes is complex and varies depending on cell types, containing varying amounts of phospholipids, cholesterol, sphingolipids, gangliosides, *etc*. In addition, lipids are not uniformly distributed, but can be organized into domains such as lipid rafts. All these factors can influence protein binding and conformation. Lipid rafts, either due to their liquid ordered phase or to the affinity of amyloidogenic proteins for specific gangliosides and cholesterol appear to be important sites for amyloid misfolding and aggregation, especially for amyloidogenic proteins involved in neurodegenerative disorders [118,119,120,121,122,123].

## 3. Amyloid Aggregation

### 3.1. The Different Steps of the Aggregation Process

**Figure 3:**
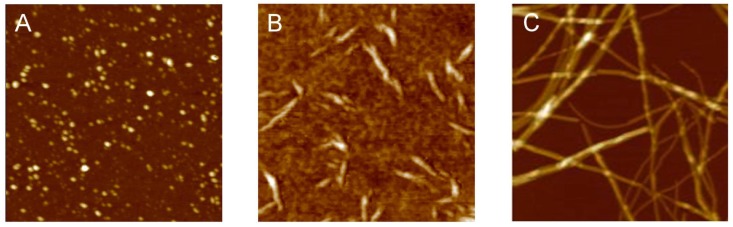
Atomic force microscopy images of representative steps of amyloid aggregation: (**A**) oligomers; (**B**) protofibrils; (**C**) mature fibrils. Scan size 1.0 µm. Z range (A) 8.0 nm; (B) 15 nm; (C) 20 nm. Relini, unpublished results.

Many different proteins, each one endowed with a specific native structure encoded in its amino acid sequence, as well as IDPs, have been reported to form amyloid or amyloid-like fibrils. Fibrils are formed through different steps. Protein monomers self-assemble into oligomers (Figure 3A), aggregates with globular shapes formed by a few monomer units. Oligomers self-assemble into more complex intermediate structures, with linear or closed-loop shape, named protofibrils (Figure 3B). The latter finally evolve into mature amyloid fibrils (Figure 3C), which are formed by the intertwining of protofilaments characterized by the typical cross-β architecture, with β-strands running perpendicular to the fiber axis and forming β-sheets parallel to the fibril axis. Such structure gives rise to typical reflections of 4.7–4.8 Å and 10 Å in the X-ray diffraction pattern, corresponding to the spacings between adjacent strands and sheets, respectively. This highly ordered structure, the cross-β spine, is common to all amyloid or amyloid-like fibrils and can be further stabilized by additional hydrogen bonding between the side chains [124].

The degree of structural order increases from oligomers to protofibrils and further to fibrils. For example, in α-synuclein, an increase in β-sheet content has been observed [125]. It has been suggested that protofibrils grow by a mechanism of oligomer addition and coalescence [126,127]; a conformational conversion of oligomers, corresponding to an increase in β-sheet content, may occur either before or concurrently with oligomer association into protofibrils [128]. The number of backbone amide protons unable to undergo hydrogen/deuterium exchange has been reported to increase from protofibrils to fibrils, implying increased order [129]. Protofibrils share some common structural properties with mature fibrils, such as linear morphology and the ability to interact with specific antibodies, but at the same time display a structural correlation with oligomers, sharing some common intraresidue contacts that are absent in mature fibrils, as shown by solid state NMR studies [130,131]. This suggests that protofibrils convert into fibrils through a remodeling of the β-sheet structure [132], increasing the number of residues involved in the β-strands [130].

Amyloid aggregation is a nucleation-dependent process. It is usually characterized by the presence of a lag phase, corresponding to the time required for the critical nuclei to form, followed by an exponential growth phase [1]. Stirring, shaking or ultrasonication of the aggregating solution accelerates the formation of critical nuclei, thus reducing the lag phase [133]. The addition of preformed fibril seeds can reduce or even suppress the lag phase [1,48,73,133]. Different kinetic and thermodynamic approaches have been proposed for modeling the mechanisms of protein aggregation (reviewed in [134]). It has been demonstrated that the exponential growth phase involves secondary pathways such as fibril fragmentation [135] or fibril branching [136]. It has recently been shown for Aβ(1–42) that once a critical concentration of fibrils is reached, oligomers are formed by a secondary nucleation mechanism that involves oligomer formation from monomers catalyzed by fibrils [137]. All these mechanisms increase the number of available growth sites, while fibrils exhibit a linear growth by monomer addition at one or both ends [138,139,140]. Fluorescence microscopy and atomic force microscopy measurements have shown that fibril growth occurs intermittently with long pauses between shorter growth periods [141,142]. An analysis of the distributions of stop and growth times found that the probability of being in the stopped state was three times larger than the probability of being in the growth state [141].

### 3.2. Polymorphism of Amyloid Aggregates

The process leading to fibril formation is strongly polymorphic. At a morphological level, a variety of intermediate structures have been described. Depending on the protein and on the aggregation conditions, protofibrils display a wide range of morphologies, including rod-like structures [143,144,145], beaded worm-like protofibrils [126,143,146,147,148,149,150], large crescents and ring-like structures [91,151,152,153,154,155,156,157,158,159]. The latter can be either large rings formed by many oligomeric units [151,152] or pore-like structures formed by a few oligomeric units [91,152,153,154,155,156,157,158,159]. For Aβ, these small annular protofibrils have been demonstrated to be on a pathway distinct from amyloid fibril formation, but they have been found in the brains of AD patients as well [159,160,161]; this suggests that even off-pathway structures may be associated with patho-physiological conditions. Different protofibrillar structures can also be sampled along a single aggregation path, as in the case of the protein HypF-N, with globular oligomers assembling into crescents and then into large rings, which break, evolve into ribbons and then develop into fibrils [151].

Different protofibril populations have been found to coexist at the same aggregation time. These populations exhibit similar beaded chain morphology but significantly different values of contour length, end-to-end distance and persistence length, which correspond to values of nanomechanical parameters, such as bending rigidity and elastic modulus, differing by one order of magnitude. This suggests different arrangements of the polypeptide chain in the two-protofibril populations [148].

**Figure 4:**
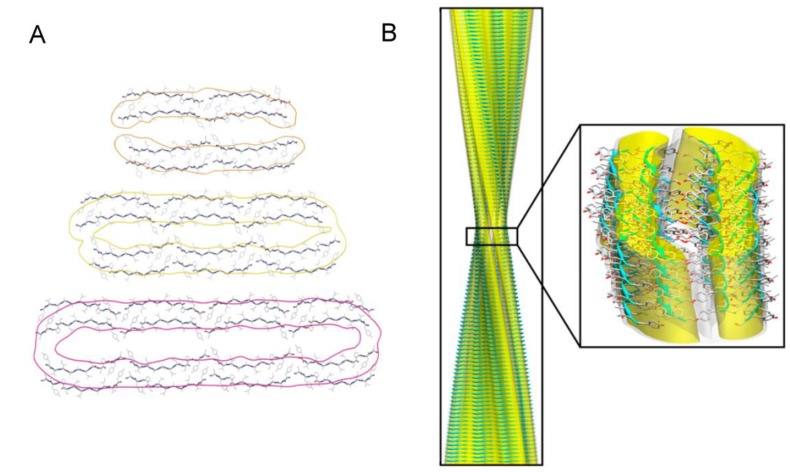
Polymorphism of TTR(105–115) amyloid fibrils. (**A**) All-atom representation of the doublet (top), triplet (middle), and quadruplet (bottom) fibril cross-sections with cryo-EM envelopes superimposed. (**B**) Atomic-resolution structure of the triplet fibril fitted into the cryo-EM reconstruction. Modified from Fitzpatrick A.W.P. *et al*., *Proc. Natl. Acad. Sci.* *USA* **2013**, *110*, 5468–5473. Copyright Fitzpatrick A.W.P. *et al*. 2013 [167].

Although mature amyloid aggregates share common features, such as the fibrillar morphology and the cross-β structure, they are characterized by a remarkable polymorphism at different structural levels. Mature fibrils result from the intertwining of a variable number of protofilaments, which gives rise to different fibrillar polymorphs, as reported for a number of proteins, including calcitonin [162], insulin [163], Aβ [164,165], glucagon [158]. Variability in the number of protofilaments constituting the fibrils has been observed even at fixed aggregation conditions [163,165,166,167]; for example, Meinhardt *et al*. have shown that Aβ(1–40) self-assembles into twelve different coexisting fibrillar structures [165]. Recently, the structures of three amyloid polymorphs formed by a 11-residue fragment of the protein transthyretin, TTR(105–115), and composed of pairs of two, three, and four interconnected protofilaments respectively, have been reported at atomic level [167] (Figure 4). A different packing configuration of the same number of protofilaments can also result in different fibrillar structures, as proposed for α-synuclein [168].

Different aggregation conditions, including the presence or absence of agitation [169,170], buffer ionic strength [171,172], temperature [158,172], and cosolvents [173,174] can result in different aggregation pathways corresponding to different aggregate structures. In addition, it has been repeatedly shown that the aggregate structural features can be propagated from one generation of fibrils to another by exposing protein monomers to aggregate seeds [169,174,175,176], thus exploiting a mechanism analogous to the transmission of prion strains.

From a structural point of view, evidence has been accumulating showing that the cross-β architecture can be achieved in many ways by the polypeptide chain, which can assume different arrangements in the amyloid fibril. The region of the chain directly involved in the cross-β structure can encompass a small or large portion of the chain, even the whole chain for small peptides, giving rise to a variety of structures [44].

**Figure 5:**
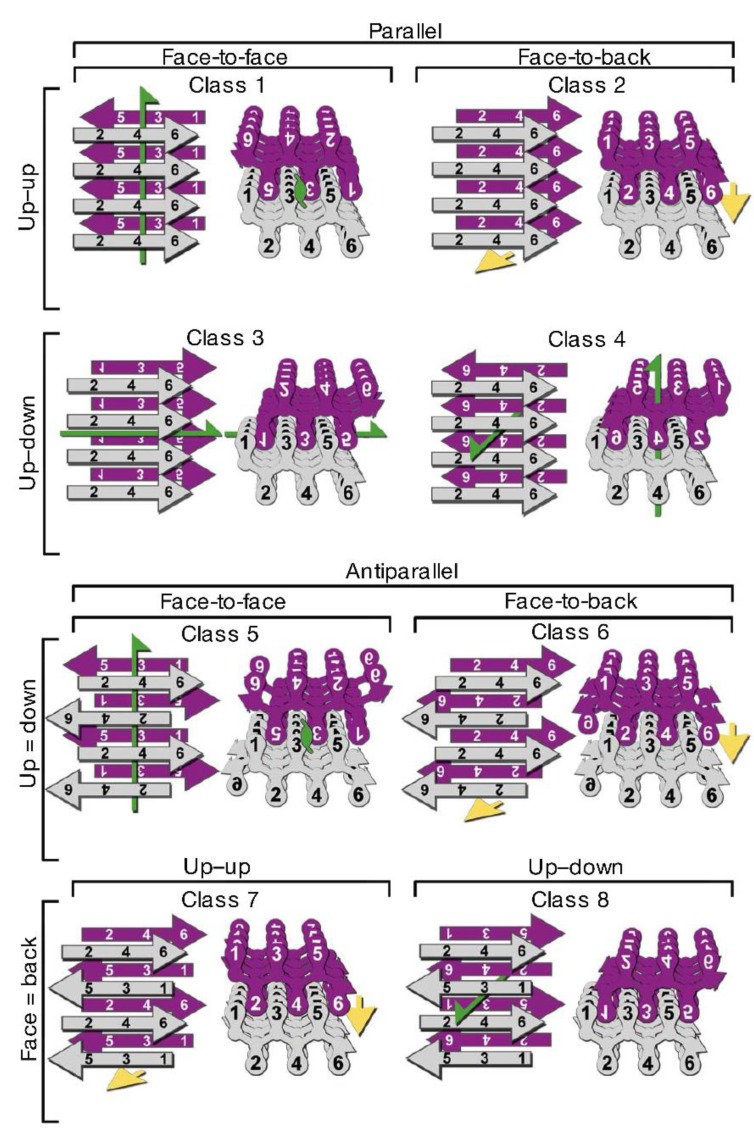
The eight classes of steric zippers (for details see text). Green arrows show two-fold screw axes, and yellow arrows show translational symmetry. Adapted by permission from Macmillan Publishers Ltd.: *NATURE*, Sawaya M.R. *et al*., Atomic structures of amyloid cross-β spines reveal varied steric zippers. 447, 453–457, copyright 2007. [178].

X-ray diffraction studies on microcrystals formed by short amyloid-forming segments have greatly contributed to the elucidation of the structure of the cross-β spine [177,178]. The identical β-sheets facing each other in the protofilament form an interface in which the highly complementary side chains from opposing sheets are interdigitated and water is excluded. Such a structural arrangement has been named a “steric zipper”. Depending on the orientation of the faces of the β-sheets (face-to-face, which is the most common, or face-to-back), on the orientation of β-strands (the same edge of the strand up for both sheets, or one up and the other down), and on the parallel or antiparallel arrangement of the strands, eight different classes of steric zippers are possible (Figure 5) [178]. Examples for all these classes have been observed experimentally for short amyloid-forming peptides [124,179]. For amyloid fibrils of full length proteins, it has been proposed that polymorphism may result either from a packing polymorphism, meaning that an amyloid segment can pack in different ways (as an example, with a shift in the interdigitation between facing sheets), or from a segmental polymorphism, involving steric zipper formation by different segments of the same protein, or from heterosteric zipper formation by non-identical β-sheets [124].

Within the protofilaments, β-strands are usually in register. A distinct amyloid aggregation pathway producing fibrils in which the strands of the contributing β-sheets are out of register has also been reported [180]. Due to the presence of unsatisfied hydrogen bonds, out-of-register fibrils are less stable and can easily convert into toxic oligomers with β-barrel structure, termed cylindrins [180,181].

### 3.3. Structural Properties of Oligomers

Oligomers represent the first assembly step in the aggregation route. When inspected by atomic force microscopy, they appear as round objects, with height varying between a fraction of a nanometer and a few nanometers [182,183,184]. In this context, the term “oligomers” refers not only to aggregates formed by a small number of molecules but is also used to describe early assemblies of a relatively large number of molecules still retaining a non-filamentous morphology [185].

The investigation of the structure of oligomeric aggregates is not an easy task, as they are transient species and are often found in coexistence with different aggregate populations. Valuable information has been obtained by X-ray diffraction [186,187,188,189], NMR [190,191,192,193], small angle X-ray scattering [194], site-directed spin labeling combined with electron paramagnetic resonance (EPR) [195]. These techniques allowed the elucidation of specific oligomeric structures, enabled the observation of similarities and differences between the structures of oligomers and mature fibrils, and demonstrated the presence of polymorphism at oligomer level.

Oligomers often have a β-sheet structure [151,182,190], but cases in which they are composed of loosely aggregated β-strands have also been reported. The latter then require strand orientation and alignment to convert from oligomers to fibrils [192]. As discussed in Section 2.1, even native-like oligomeric aggregates can be formed [7,196]. Different aggregation conditions can result in oligomers with similar size and β-sheet content but different packing of the hydrophobic groups, giving rise to different oligomer toxicities, as in the case of the non pathogenic protein HypF-N [184]. Different oligomer species have even been found to be involved in the same aggregation process [158] or to coexist at fixed aggregation times [197]. Based on the analysis of the interaction of oligomers with conformation-dependent antibodies, distinct types of oligomers have been identified. Oligomers able to react with fibril-specific antibodies, but not with antibodies specific for other oligomers, were named “fibrillar oligomers”, as they shared a common epitope with mature fibrils; a second type of oligomers was named “prefibrillar oligomers” as they were not able to interact with fibril-specific antibodies, but reacted with oligomer-specific antibodies [183,197]. This suggests different aggregation mechanisms, as prefibrillar oligomers should undergo a conformational change before evolving into fibrils, while fibrillar oligomers can act as a template for the addition of new monomers. Recently, it has been demonstrated that fibrillar oligomers of Aβ(1–42) have a cross-β architecture [198] supporting this view.

It is not surprising that polymorphism occurs in oligomers. Not only can it reflect the coexistence of aggregates with slightly different maturation stages, but it also presages the variety of structures that the mature fibrils can assemble into. Moreover, it can result from the alternative aggregation paths from the monomer to mature fibrils (Reviewed in [199]). In addition, oligomer polymorphism with related differences in toxicity could have interesting implications *in vivo*, as it could explain the presence of large fibrillar deposits in healthy subjects [197].

## 4. Amyloid Aggregates Disrupt Membrane Integrity

Increasing evidence indicates that the interaction of amyloid aggregates with membranes is critical in the onset and progression of amyloid diseases. Why are amyloidogenic peptides toxic to cells? It is commonly accepted that the interaction between amyloid aggregates and cell membranes, either the plasma membrane and/or membranes of internal organelles results in disruption of intracellular calcium homeostasis and oxidative stress. In Alzheimer’s disease Aβ aggregates can change synaptic plasticity and induce neuronal cell death. In most cases, oligomers rather than mature fibrils, are the toxic species that disrupt the membrane permeability. However, in some cases, mature fibrils may also induce cell damage disassembling the membrane lipids [14,19,200,201]. So far, the major unsolved problem remains the molecular mechanism whereby the barrier properties of the cells are reduced, although many models have been proposed (reviewed in [11]). In what follows, we report recent experimental findings, suggesting that more than one mechanism might be active in increasing the ionic fluxes, which cause interference with ion homeostasis and the toxic cascade of events associated with amyloid diseases.

### 4.1. Formation of Ionic Pores

**Figure 6:**
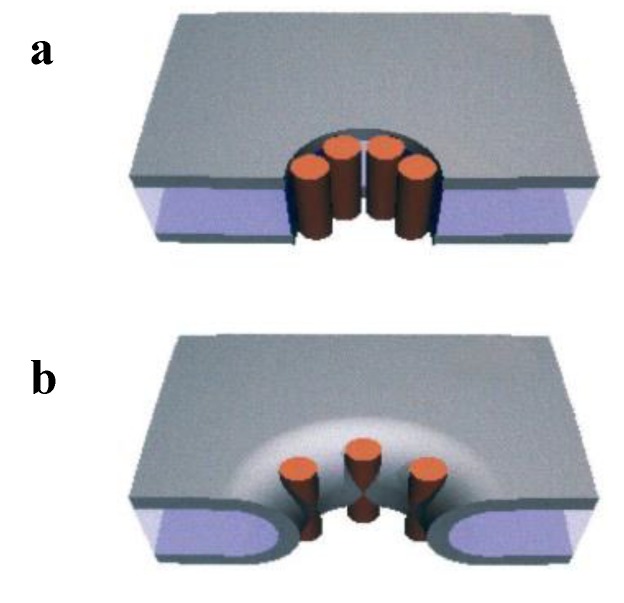
Two possible models for pores (**a**) Barrel-stave pore; (**b**) Toroidal pore. Reprinted from *The Biophysical Journal*, **81**, Yang, L.; Harroun, T.; Weiss, T.; Ding, L.; Huang, H. Barrel-stave model or toroidal model? A case study on melittin pores, 1475–1485. Copyright 2001 with permission from Elsevier [203].

Formation of protein pores, which allow the passage of ions is one obvious mechanism for amyloid aggregate toxicity. The ground-breaking work proposing that amyloid aggregates form well defined channels in the membrane was performed by Arispe and coworkers in 1993 [202]. Using electrophysiological techniques applied to planar lipid bilayers (BLMs), these researchers found that amyloid aggregates of Aβ(1–40) were able to induce cation selective single channel currents, reminiscent of those of ion channels found in cell membranes. Since then, many other amyloidogenic proteins, including hIAPP, α-synuclein, and prion proteins have been shown to form ionic channels in artificial membranes as reviewed by Butterfield and Lashuel [11]. Protein pores can be modeled in two ways, barrel stave pores such as those forming ion channels (Figure 6, top) or toroidal pores formed with both protein and lipid components (Figure 6, bottom) [203].

Given the large number of reports in this field, it seems difficult to provide a global picture on pore formation by the different proteins responsible for amyloid diseases; we focus our attention on recent studies that shed new light on mechanisms and consequences of Aβ oligomer interactions with membranes.

An interesting oligomeric form, the annular Aβ protofibril, has been suggested to be responsible for membrane permeability. Based on its pore-like structure it has been proposed that it produces membrane permeabilization [156]. Surprisingly, annular protofibrils interacting with BLMs exhibited a much lower membrane permeabilizing activity than their corresponding amyloid precursors. It has been proposed that preformed annular protofibrils in solution do not insert into the membrane efficiently, while oligomers can assemble on the membrane and give rise to a membrane-embedded pore [159]. These structures may be physiologically relevant, as annular protofibrils have been shown to be intracellular structures present in Alzheimer’s disease brains [160,161].

Subsequent studies have pointed out the enormous variability of the pore conductance levels and have suggested that pores are dynamic structures formed by assembly and disassembly of oligomers without a fixed stoichiometry. In particular, it has been shown that the interaction of Aβ with the membrane and the capability to form ionic channels with well-defined conductance states critically depends on peptide concentration in the bathing solution [115,116]. Combining single molecule fluorescence microscopy with ionic recordings through BLMs, Schauerte *et al*. [115] showed that hexamers were the lowest oligomeric species of Aβ(1–40) able to permeabilize the membrane. Importantly, they worked in the nanomolar concentration range, which is close to that observed in the human brain [204]. At concentrations on the order of mM they found the largest conductivity for ions corresponding to 100 pS/pore [115]. Large variability in conductances, attributed to the different stoichiometries of the Aβ oligomer aggregates, was also measured in patch clamped *Xenopous laevis* oocytes [205]. Exploiting high-resolution optical imaging, the authors recorded Ca^2+^ influx due to Aβ(1–42) oligomers [159,205]. The currents are blocked by Zn^2+^ ions, implying that specific protein structures with defined pores are involved. The results provide support for a mechanism whereby amyloid oligomers in the range from five-to 40-mers directly form Ca^2+^ permeable pores. Based on experiments and modeling, Stroud *et al*. proposed transmembrane channels with a well defined structure formed by Aβ(1–42) fibrillar oligomers with a cross-β structure [198]. It appears that even shorter fragments of Aβ can form ionic channels that allow Ca^2+^ uptake and are blocked by Zn^2+^ ions. Jang *et al*. have found that channels can also form from the non-amyloidogenic truncated Aβ fragments Aβ(11–42) and Aβ(17–42), which are found together with Aβ(1–40) and Aβ(1–42) in the amyloid plaques of AD [206]. The electrical activity recorded from these structures, formed by loosely attached subunits, provides support for the idea that the channels are dynamic structures that are formed and disrupted according to the oligomer aggregation-disaggregation process.

Experiments on ion fluxes have not necessarily been interpreted as showing discrete protein channels. In experiments performed under well-controlled aggregation condition over 20 days, several parameters including pore formation, the size of Aβ(1–40) or Aβ(1–42) oligomers, and cytotoxicity in human neuroblastoma cell lines were correlated. The results showed that only oligomers in the range of tetramers to 13-mers induced Zn^2+^-inhibited ionic fluxes through BLMs, while smaller and larger oligomers did not [207]. Again the authors found a large variety of conductance levels of the recorded currents. Discussing the permeation mechanism, they considered it equally possible that either trans-membrane protein channels or defects, due to a mechanism similar to that of antimicrobial peptides (see later), were formed, or even that both mechanisms acted in parallel.

In conclusion, there are several reasons that might explain discrepancies in results from different laboratories, especially in early studies. First of all, as already reported in previous sections and illustrated in Figure 2, oligomers are polymorphic and consist of a dynamically distributed ensemble of aggregates that are difficult to stabilize and fractionate in pure forms [44,182,207]. Moreover, different structures form under different conditions. Finally, lipid composition can also play a role in the aggregate-membrane interaction [199]. Thus, a wide variety of aggregated forms of Aβ peptides induce ionic flows through artificial and natural membranes, probably acting with more than one permeation mechanism.

### 4.2. Tension-Induced Poration Mechanism

The tension-induced poration mechanism, which has been used to describe the action of antimicrobial peptides, can be applied to poration by amyloids as well. Antimicrobial peptides lead to cell death by disrupting the microbial membrane. The mechanism has been the object of studies for several years [208,209,210,211,212,213]. As is the case for amyloids, it has been proposed that these peptides act through the formation of pores. A very interesting model based on the notion of membrane tension was introduced by Huang *et al*. [209]. Peptides, characterized by amphipathic regions, can bind to the lipid bilayer in the region between polar heads and acyl chains, altering the energy landscape. Such binding produces an area expansion, which in turn causes both membrane thinning and internal stress (or membrane tension), due to the unfavorable packing of the acyl tails. Forming pores increases the available exposed surface, thus membrane tension is released and the initial thickness can be restored. The pores were modeled either as stable and well defined peptide-lined structures [208] such as a barrel stave pore, created by alamethicin [214] or as a toroidal pore, such as that proposed for magainin or the venom melittin peptides [203] (see Figure 6) or, more recently, as a transient stochastic-type channel, created by structural distortions involving both lipids and peptides [212,215]. Kinetic studies performed on artificial liposomes exposed to cecropin A [216], magainin 2 [211,212], and melittin [217] have shown that the membrane tension model provides a quantitative explanation of the experimental findings without involving any specific arrangement of these peptides.

Recent work has applied this model to amyloidogenic proteins. Studies exploiting single molecule techniques, performed exposing liposomes to IAPP have shown that at very low concentrations (in the nanomolar range), the membrane-peptide interaction is due to the stochastic nucleation of oligomers on the membrane and does not cause leakage. Upon addition of further peptides, the oligomeric state expands and then evolves into a leaky state due to pores, which are stable for days [218]. The poration mechanism is described through the formation of an intermediate non-amyloid protein/lipid species resulting in all-or-none leakage. The authors have suggested that the membrane tension model is one possible explanation for these results. Further work has shown that the simultaneous action of magainin 2 and IAPP enhances both the fluxes though liposomes and the bacterial growth inhibition by more than hundred-fold, a cross-cooperativity much higher than the simple sum of the actions of the individual peptides [216]. This body of results argues in favor of the hypothesis that antimicrobial peptides and amyloids share a common poration mechanism. Membrane permeability would result from pores formed by dynamically sized oligomers, due to a nucleation dependent phenomenon, described by a model including the membrane tension concept.

### 4.3. Disassembly of the Lipid Bilayer: Role of Oligomers and Fibrils

**Figure 7:**
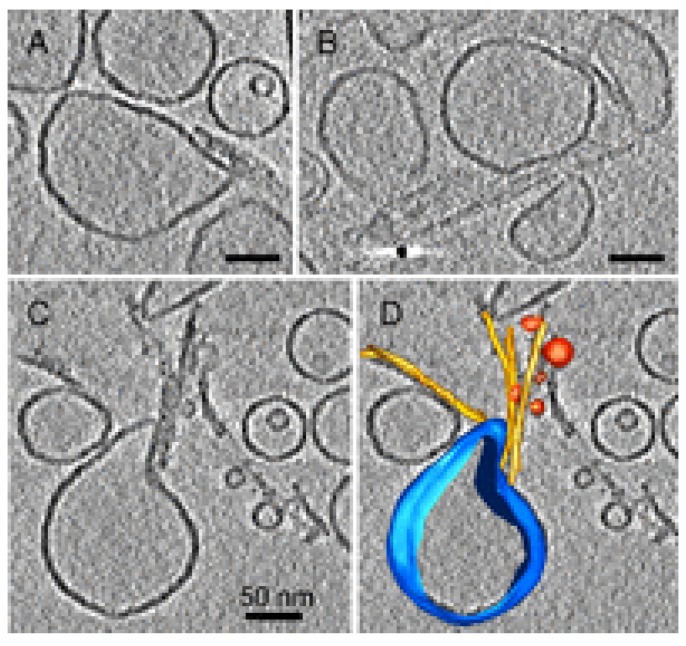
Distortion of liposomes caused by fibril of β2-microglobulin visualized by cryoelectron tomography (**A**–**C**) sections of tomogram; (**D**) rendered 3D model of (C). (C,D) also show blebbing. Modified from Milanesi, L. *et al*., *Proc. Natl. Acad. Sci.* *USA* **2012**, *109*, 20455–20460. Copyright Milanesi, L. *et al*. 2012 [200].

Experimental results have supported bilayer disassembly as an additional disruptive mechanism. Atomic force microscopy (AFM) studies on sBLMs exposed to HypF, either in the monomeric or in the oligomeric aggregation state, have shown that the amyloid-like peptide induces disorganization of bilayer resulting in membrane defects and/or thinning. The disruption of the lipid organization reduces the barrier to ion transport [13,219]. Valincius *et al*. performed work aimed at assessing the molecular mechanism of ion transport in artificial membranes exposed to Aβ oligomers [220]. They exploited a variety of techniques, including neutron reflectivity from tethered sBLMs, capacitance and conductance measurements. Their results were not compatible with bilayer-spanning pores but were better described by a model in which oligomer insertion into the lipid bilayer produces a local increase in the dielectric constant. This would reduce the barrier properties of the bilayer, causing ion permeability. In addition to the previously proposed role of oligomers, a direct role of fibrils in membrane impairment has been described, and various mechanisms come into play. Priming membrane leakage might involve fibrils of reduced stability as sources of toxic oligomers that would act as already described [14]. Alternatively, amyloid fibrils, growing at membranes, might extract lipids by interacting with the outer leaflet. Sparr *et al*. first described the uptake of lipids induced by hIAPP interacting with liposomes during amyloid fibril formation and the associated loss of the barrier properties of the membrane [221]. A subsequent work showed that hIAPP fibrils grew on membranes and lined the surface of distorted lipid vesicles. Comparing the similarity of the kinetic profiles of thioflavine T fluorescence and dye leakage, the authors propose that fibril growth is connected with membrane damage through a mechanism that also involves changes in membrane curvature and in lipid packing [19]. A similar behavior was described by Milanesi *et al*. [200] in experiments performed exposing liposomes to β2-microglobulin fibrils formed *in vitro*. The results showed that fibril tips interact with lipid membranes and cause distortions in the liposome shape (see Figure 7). Moreover, fibrils extract lipids from the membranes at the points of interaction, thus causing blebbing and damage to the bilayer organization (see Figure 7C,D).

Other examples highlighting the importance of fibrils in their interactions with lipid membranes have been supported by experimental results. Pieri *et al*. assessed the interaction of fibrillar α synuclein and Hungtingtin Exon 1 with cultured cells and lipid vesicles [201]. They found that homogeneous populations of fibrils, much more than their precursors, produced permeabilization of the membrane and intracellular Ca^2+^ increase, causing cell death. However, according to the authors, the molecular mechanism of such interaction, which seems to depend on lipid composition, is still unclear. Artificial out-of register amyloid mimics produced fibrils that are toxic to PC12 cells [180], and fibrils grown from the yeast prion Sup35p were also found to damage cultured cells [222]. However, at variance with the aforementioned cases, cell exposure to fibrils of Sup35p did not produce any increase in membrane permeability, instead, an abnormal accumulation of raft domains was observed. The authors propose that such raft assembly plays a key role in cell impairment.

It is also possible that different segments of the same protein play different roles in inducing membrane damage. For instance, it has been proposed that hIAPP impairment to cells consists of two different processes related to two distinct segments [223]. Firstly, the N-terminal fragment 1–19, which does not form amyloid fibers, inserts and interacts with negatively charged phospholipids [224]. This fragment produces the early membrane disruption of β-cells [225] and causes dye leakage in liposomes [226], possibly with a pore-like mechanism, while the 20–29 region favors amyloid aggregation [224] and causes membrane disassembly, induced by fibril growth at the membrane [19]. Using solid-state NMR experiments, Brender *et al*. have shown that lipid disorder caused by the 20–29 fragment likely plays a minor role in membrane disruption and toxicity [223].

In summary, the experimental results reported above show that not only oligomers but fibers also affect the organization of the membrane and can give rise to cell impairment.

### 4.4. Oligomer Interaction with Specific Cell Structures

Much of the experimental data so far reported uses model systems such as BLMs or liposomes. However, the scenario is much more complex in biological membranes. There are specific mechanisms of cell impairment, caused by amyloid peptides, which cannot be reproduced easily in model systems. In this section, we report examples of investigations that describe the interactions of amyloid aggregates with membrane receptors, lipid rafts, and nuclear membranes.

Exogenous Aβ oligomers bind at nearby synapses and modulate the properties of membrane receptors exploiting several mechanisms. They were shown to bind to or in close proximity of ionotropic glutamate receptors, namely N-methyl-D-aspartate (NMDA), causing detrimental Ca^2+^ influx in hippocampal neuronal cultures [16,227]. Moreover, they can cause synaptic dysfunction through endocytosis of NMDA receptors [228]. Deleterious impairment of neurons and astrocytes was also induced by binding of Aβ oligomers at membranes that caused diffusional trapping and clustering of a metabotropic receptor (mGluR5) [229]. In our own laboratory, we found that amyloid oligomers of HypF-N interacts with glutamate receptors of rat cerebellar granule cells inducing a transient increase of Ca^2+^ permeability and cytotoxicity [230]. In a subsequent work, Tatini *et al*., showed that toxic HypF-N oligomers caused synaptotoxicity, while the control non-toxic conformer produced none of the toxic effects [231]. The distinct mechanisms by which a toxic oligomer can affect cell permeability have been demonstrated by experiments performed exposing rat cerebellar granule cells to early amyloid oligomers of an expanded Ataxin 3 variant [17]. The interaction with the membrane caused neuronal damage through Ca^2+^ influx from the medium. Colocalization experiments indicated that the interaction involved glutamate receptors, voltage-gated channels, and ganglioside-rich membrane domains. In contrast, the interaction with a more aged pre-fibrillar aggregate of the same protein caused Ca^2+^ permeation by a mechanism involving only ganglioside-rich areas [17]. This result underlines the importance of the different amyloid conformations in determining the distinct and non–overlapping mechanisms by which Ca^2+^ influx and neurotoxicity occur, consistent with what was observed for Aβ oligomers [232]. In addition to the amyloid conformation or the specific amyloid fragment [233], membrane lipid composition and the presence of lipid rafts in the plasma membrane were shown to affect bilayer stability and cell vulnerability to the amyloidogenic protein [15,234].

Receptors affected by amyloidogenic proteins need not be those involved directly in neurotransmission. Familial amyloidotic polyneuropathy is a pathology characterized by systemic extracellular deposition of mutant transthyretin (TTR) amyloid fibrils. It has been shown that binding of TTR fibrils to the receptor for advanced glycation end products (RAGE) causes dysfunction in the peripheral nervous system [235] and alters signal transduction [236]. In the Alzheimer’s disease brain, an inflammatory pathway is triggered by interaction of Aβ peptides with membrane bound RAGE [237], while the binding of soluble Aβ to soluble RAGE inhibits further aggregation of Aβ peptides [238]. Recently, it has been found that a leukocyte immunoglobin-like receptor, present in the human brain, binds to Aβ oligomers with nanomolar affinity and may contribute to synaptic loss [18].

So far we have described cellular impairment caused by the interaction of the cell membrane with amyloid peptides. However, not all amyloid peptides produce extracellular amyloid deposits. For instance, Ataxin 1, which causes spinocerebellar ataxia type 1 when associated with an uninterrupted polyglutamine expansion, accumulates within the cell. Therefore, experiments were performed to check whether intracellular structures were damaged. Electrophysiological measurements on the inner nuclear membrane of cells transfected with the pathological variant of Ataxin 1 revealed the appearance of abnormal ionic fluxes. These are caused by dynamic pores, which are created due to the interaction of the aberrant protein with the nuclear membrane [239].

In summary, the above experimental findings shed a glimmer of light on the still unsolved problem of the specificity of damage of the different peptides in amyloid diseases. In fact, membrane receptors, lipid composition, and lipid rafts that are specific to cells in a particular tissue may be the sites of noxious interactions with amyloid oligomers.

## 5. Conclusions

In this review, we discussed how defects in folding of some proteins produce amyloid fibers, which deposit in various tissues, and are associated with a number of serious illnesses. The exact pathway from the first misfolding to the onset of disease is still not entirely clear. To further this understanding, it is important to analyze the conformational states (unfolded, partially folded or native-like) and environmental conditions that lead to aggregation as well as mutations that favor this process. Oligomers, protofibrils, and fibrils are polymorphic, and their toxicity varies depending on the structure of the aggregates, even for the same protein [184]. An increasing number of studies have highlighted the role of membranes and their lipid composition in influencing the aggregation process. Aggregation at the membrane often results in lipoprotein assemblies that favor insertion into the membrane and can result in disruption of membrane function. There is general agreement that oligomers are responsible for the cascade of events leading to cytotoxicity, but fibrils, when they grow on membranes, can extract lipids and thereby also cause damage. On the other hand, there is no agreement on the molecular mechanisms for oligomer-induced membrane damage and disruption of cellular homeostasis. Several models have been proposed: protein pores, membrane thinning, protein-induced membrane defects, and interactions with receptors or other cellular components. These models are not mutually exclusive, oligomers can act in more than one way. The mechanism observed can be influenced by several factors: the aforementioned oligomer polymorphism, different chemical/physical properties of oligomers that seem morphologically similar, aggregate concentration, potential differences of oligomers prepared in different laboratories, cell type and membrane composition. These differences lead to different vulnerability of cells to pathological proteins. An added complication is shown by surprising, and thus far unexplained, studies that show that oligomers prepared from patients can have cytotoxic effects similar to synthetically prepared oligomers but at two or three orders of magnitude lower concentrations [204]. Thus more in-depth comparison of oligomers formed *in vitro* or *in vivo* is needed.

We have attempted to highlight the complexity of the situation. A multifactorial analysis seems the most promising for furthering understanding and thus aiding in the development of drugs to prevent initiation and progression of amyloidogenic diseases. Cytotoxicity can be associated with different stages and conditions of oligomer formation and can vary depending on the cell type with which the oligomers interact. Such an analysis should seek to correlate these variables with specific interactions with different membrane components, lipid and protein, extra- and intra- cellular, that are present *in* *vivo*.
